# Effects of functional performance and national health insurance cost on length of hospitalization for postacute care in stroke: a retrospective observational study

**DOI:** 10.1186/s12883-023-03396-z

**Published:** 2023-09-29

**Authors:** Hsiang-Yun Chou, Shang-Chun Ma, Ya-Wen Tsai, Chia-Li Shih, Chieh-Ting Yeh

**Affiliations:** 1 Department of Rehabilitation, An Nan Hospital, China Medical University, No. 66, Sec. 2, Changhe Rd., Annan Dist, Tainan, 709204 Taiwan; 2 Institute of Physical Education, Health & Leisure Studies, National Cheng Kung University, No. 1, Daxue Rd., East Dist, Tainan, 701401 Taiwan; 3 Department of Nursing, An Nan Hospital, China Medical University, No. 66, Sec. 2, Changhe Rd., Annan Dist, Tainan, 709204 Taiwan

**Keywords:** Stroke, Postacute care, Disability, Healthcare costs, Health policy, Decision making, Health care allocation

## Abstract

**Background:**

The postacute care for cerebrovascular disease (PAC-CVD) program was launched in Taiwan nearly a decade ago. However, no clear regulations regarding length of stay (LOS) in the program and extension standards exist. Thus, the allocation of limited medical resources such as hospital beds is a major issue.

**Methods:**

This novel study retrospectively investigated the effects of functional performance and national health insurance (NHI) costs on PAC-CVD LOS. Data for 263 patients with stroke who participated in the PAC-CVD program were analysed. Hierarchical multiple regression was used to estimate the effects of functional performance and NHI costs on LOS at three time points: weeks 3, 6, and 9.

**Results:**

At week 3, age, NHI costs, modified Rankin scale score, and Barthel index significantly affected LOS, whereas at week 6, age and NHI costs were significant factors. However, functional performance and NHI costs were not significant factors at week 9.

**Conclusions:**

The study provides crucial insights into the factors affecting LOS in the PAC-CVD program, and the results can enable medical decision-makers and health care teams to develop inpatient rehabilitation plans or provide transfer arrangements tailored to patients. Specifically, this study highlights the importance of early functional recovery and consideration of NHI costs when managing LOS in the PAC-CVD program.

## Background

According to the World Health Organization, stroke is the second leading cause of death worldwide and the third leading cause of disability [1]. In the United States, over 795,000 strokes are reported annually, of which approximately 610,000 are first-time occurrences [2]. In Taiwan, which has a population of approximately 23 million people, approximately 80,000 first-time or recurrent stroke events occur annually, and the medical expenses related to stroke are estimated at US$375 million. Each patient with stroke spends approximately US$32,367 on stroke-related medical care over their lifetime [3]. Studies have demonstrated that during the 6–10 weeks following a stroke, an estimated 16–42% of the body functions and activities undergo spontaneous recovery [4]. Additionally, earlier rehabilitation after stroke is associated with greater improvements in functional outcomes [5].

Regarding treatment to recover function or reduce disability and alleviate the economic burden incurred by stroke, the National Health Insurance Administration (NHIA) of Taiwan launched a nationwide postacute care for cerebrovascular disease (PAC-CVD) program in 2014. This program gives patients high-intensity inpatient rehabilitation and integrated care implemented by a multidisciplinary PAC-CVD stroke team [6]. Studies have reported that the PAC-CVD program has significantly improved the physical function, activities of daily life, nutritional status, cognitive function, and quality of life of patients with stroke [7–11]. The PAC-CVD program sustainably improves patients’ physical function and walking ability over 12 weeks of hospitalization. Medical expenses are controlled through the NHI per-diem reimbursement system (NT$3645 per day) and submission of hospitalization extension applications [12]. However, in clinical practice, the allocation of medical resources such as length of stay (LOS) given limited bed capacity must be analysed. When a hospital lacks an available PAC-CVD bed, a patient must wait for an available bed or be transferred to a hospital that does have such a bed available [13]; this results in the suspension of rehabilitation and potentially affects recovery of physical function. Taiwan has offered the PAC-CVD program for approximately 9 years, but the NHIA has no clear criteria for defining LOS and the extension of PAC-CVD. Under the per-diem reimbursement system, longer hospitalization leads to higher NHI costs and may also prevent patients with relatively acute disease from receiving PAC-CVD program treatment due to a lack of beds. However, shortening the LOS may affect the rehabilitation process because patients may be discharged before they have recovered their physical functions. Regaining the ability to independently walk is usually the main goal of patients with stroke [14]. Therefore, this study investigated the effect of functional performance and NHI costs on the LOS of patients with stroke participating in the PAC-CVD program.

## Methods

### Study design and patients

This study was a retrospective observational study based on medical record review. Data were collected for patients with stroke who participated in the PAC-CVD program at a regional hospital in southern Taiwan from January 2017 to June 2021. The exclusion criteria were unexpected termination of the PAC-CVD program for the patient due to a change in their medical condition (e.g., recurrent stroke, acute kidney failure, acute myocardial infarction, and death) or personal reasons and the inability to continue the program. The study protocol was approved by the Research Ethics Committee of Taiwan Municipal An-Nan Hospital—China Medical University (TMANH110-REC007). The requirement for informed consent was waived because of the retrospective design of the study. Data on patients’ demographics (i.e., age and gender), LOS, NHI costs, functional performance, clinical attributes (i.e., stroke history, stroke type, hypertension, coronary artery disease, diabetes mellitus, and hyperlipidemia), and common risk factors (i.e., smoking and drinking) were collected from electronic medical records. To retain patients’ privacy, the data were kept confidential and protected, and patients were anonymized. Once data collection was complete, statistical analysis was conducted.

### PAC-CVD program

The PAC-CVD program is the first structured PAC program implemented in Taiwan. When experiencing an acute stroke, a patient receives acute medical intervention at a medical centre or large-scale hospital. Within 1 month of their medical condition stabilizing, the patient is transferred to a community hospital (i.e., a regional or district hospital) for the inpatient PAC program. The inclusion criteria for the PAC-CVD program are: (1) experiencing an acute stroke within the preceding 30 days; (2) having stable vital signs and a stable neurological status for at least 72 h without uncontrolled complications; (3) having moderate-to-moderately severe functional impairment with a modified Rankin scale (mRS) score of 3 to 4; (4) demonstrating sufficient physical ability to sit in a wheelchair or at the bedside for at least 1 h; (5) possessing basic cognitive ability to understand rehabilitation activities and actively participate in rehabilitation therapy; and (6) having good family support.

The services provided by the PAC-CVD program include: (1) tailored treatment plans; (2) integrated care from a multidisciplinary team of clinicians, nurses, physical therapists, occupational therapists, speech therapists, social workers, dietitians, and case managers; (3) high-intensity rehabilitation (3–5 sessions per day); (4) lessons on homecare skills to prepare for discharge; (5) prevention and treatment of comorbidities and complications; and (6) regular assessments (at the beginning of hospitalization, discharge, and once every 3 weeks during hospitalization) to facilitate the development of a care plan.

The LOS is generally 3–6 weeks. If a patient requires hospitalization for > 6 weeks, the multidisciplinary team’s conference records are sent to the NHIA, which decides whether and for how long the extension can be permitted. The LOS can be extended to be 12 weeks maximum. If an extension request is rejected, the multidisciplinary team must inform the patient and plan for their discharge or a transfer of services.

### Functional outcome measures

mRS and Barthel index (BI) scores were collected to reflect general physical activity. The mRS is widely used by clinical professionals as a general disability assessment to evaluate the degree of required daily living assistance of patients with stroke and their walking ability. The scores range from 0 to 6, with 0, 1, 2, 3, 4, 5, and 6 corresponding to no symptoms, no significant disability, slight disability, moderate disability, moderately severe disability, severe disability, and death, respectively. The higher the score, the more severe is the degree of disability [15]. The BI is used to assess functional impairment in activities of daily living for patients with stroke [16]. The BI consists of 10 items, including items on feeding, bathing, grooming, dressing, bowel and bladder control, toilet use, transfer and mobility, and stair use. The total scores range from 0 to 100, with scores of 0–20 indicating complete dependence, 21–60 severe dependence, 61–90 moderate dependence, 91–99 mild dependence, and 100 no dependence. A higher score indicates greater independence in performing daily activities [17, 18].

Regarding walking ability, data were collected using the 5-m walking speed (5MWS) and the 6-min walking distance (6MWD) tests. The 5MWS is the time it takes for a patient with stroke to walk 5 m at a comfortable high speed (in m/s) on a flat surface without assistance. The 5MWS is used to evaluate both walking speed and ability [19]. The 6MWD is the total distance (in m) that a patient can walk in 6 min on a flat surface without personal assistance. The 6MWD was originally designed as a submaximal exercise test for patients with cardiopulmonary diseases [20]. Subsequently, it has been used to measure improvements in and the effectiveness of functional walking in patients with stroke after physical therapy and is commonly used in clinical settings to assess walking endurance [21].

### Statistical analysis

Patients’ gender, clinical factors (i.e., stroke type, stroke history, hypertension, coronary artery disease, diabetes mellitus, and hyperlipidemia), and common risk factors (i.e., smoking and drinking) were coded as categorical variables. Age, LOS, mRS score, BI, 5MWS, 6MWD, and NHI costs (in 2020, US$1 = NT$29.6) were coded as continuous variables and are presented as the mean and standard deviation (SD).

Hierarchical multiple regression was conducted to investigate the effect of functional performance and NHI costs on LOS in the PAC-CVD program at different time points (weeks 3, 6, and 9). Because individual factors and differences in recovery may lead to different prognoses for functional performance, patient demographics (i.e., age and gender), clinical factors, and common risk factors were set as control variables. Each regression model consisted of the following four components, which were added in sequence: (1) age and gender; (2) clinical and common risk factors; (3) NHI costs; and (4) functional performance. The dependent variable was LOS in the PAC-CVD program. Normality was examined before each regression was conducted; the absolute value of skewness should be below 2, and the absolute value of kurtosis should be below 7 [22]. To verify multicollinearity in each regression, the variance inflation factor (VIF) was used for diagnosis. A VIF value of > 5 indicates the presence of multicollinearity that must be addressed [23].

Statistical analyses were conducted using SPSS (version 26.0; IBM, Armonk, NY, USA). All tests were two-sided, and a *p* of < 0.05 indicated statistical significance.

## Results

### Patient characteristics

**Fig. 1:**
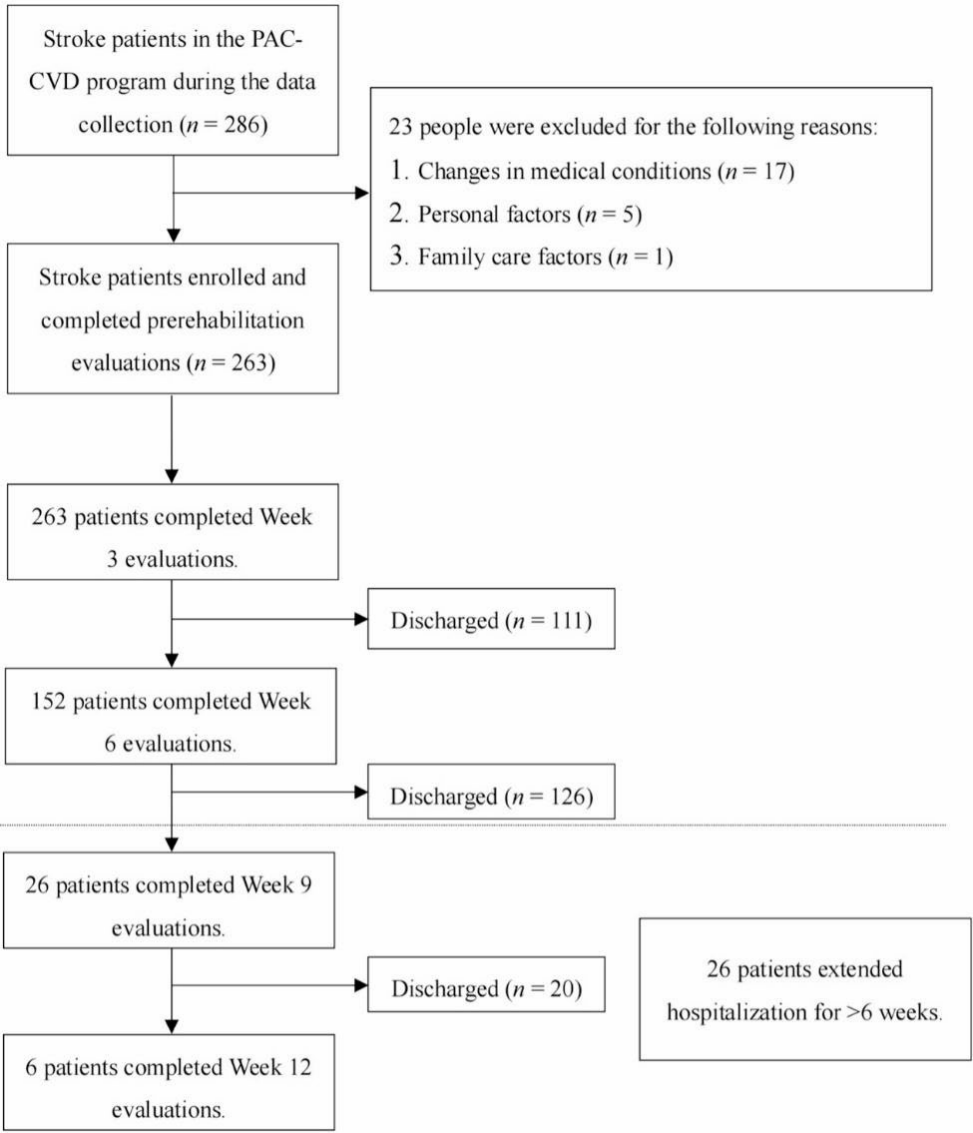
Flowchart of patient recruitment and data collection

During the period of data collection, 286 patients with stroke participated in the PAC-CVD program, and participation in the program was terminated for 23 due to changes in their medical conditions or personal factors. A total of 263 patients were thus enrolled in this study. Functional performance measurement and NHI cost data were collected at weeks 3, 6, 9, and 12 of hospitalization for 263, 152, 26, and 6 patients, respectively (Fig. 1). As shown in Table 1, the mean age of the patients with stroke was 63.74 (SD: 13.08) years. A total of 172 patients were men (65.4%), and 91 were women (34.6%). Ischemic stroke was the dominant type of stroke (71.5%); 20.2% of the patients had a history of stroke, and the proportion of patients with hypertension was 93.9%. The most common LOS was 4–6 weeks (47.9%). A total of 26 (9.9%) patients had an LOS of > 6 weeks. The mean PAC-CVD LOS was 32.57 (SD: 15.28) days. The mean magnitude of NHI costs was US$3,189.95 (SD: US$1,501.07).

**Table 1:** Patient characteristics (*N* = 263)

Variables	Mean (*SD*) or *n* (%)
Demographics	
Age, years	63.74 (13.08)
18–64	135 (51.3%)
65–84	119 (45.3%)
> 85	9 (3.4%)
Gender	
Male	172 (65.4%)
Female	91 (34.6%)
Clinical Attributes	
Stroke type	
Ischemic	188 (71.5%)
Hemorrhagic	75 (28.5%)
Previous Stroke	53 (20.2%)
Hypertension	247 (93.9%)
Coronary Artery Disease	76 (28.9%)
Diabetes Mellitus	112 (42.6%)
Hyperlipidemia	121 (46.0%)
Common Risk Factors	
Smoking	59 (22.4%)
Drinking	29 (11.0%)
Description of Hospitalization	
Weeks of Hospitalization	
0–3 weeks	111 (42.2%)
4–6 weeks	126 (47.9%)
7–9 weeks	20 (7.6%)
10–12 weeks	6 (2.3%)
PAC-CVD LOS, day	32.57 (15.28)
Cost of NHI, USD	3,189.95 (1,501.07)

SD: standard deviation; PAC-CVD: postacute care for cerebrovascular disease; LOS: length of stay; NHI: national health insurance

### Functional performance before rehabilitation

The mean mRS score before participation in the PAC-CVD program was 3.66 (SD: 0.47). Of the 263 patients, 89 patients (33.8%) had an mRS score of 3, and 174 (66.2%) had an mRS score of 4. Thus, more than half of the participants had moderate-to-severe disability and required assistance with walking and daily activities. The mean BI was 44.56 (SD: 22.23), indicating that many patients had considerable dependence for daily activities. Regarding walking speed, the mean 5MWS was 0.1 (SD: 0.24) m/s. Regarding cardiorespiratory endurance and walking ability, the mean 6MWD was 25.67 (SD: 67.64) m (Table 2).

**Table 2:** Functional performance before rehabilitation (*N* = 263)

Variables	*n* (%)	Mean (*SD*)
mRS		3.66 (0.47)
mRS 3	89 (33.8%)	
mRS 4	174 (66.2%)	
BI		44.56 (22.23)
5MWS		0.1 (0.24)
6MWD		25.67 (67.64)

mRS: modified Rankin scale; BI: Barthel index; 5MWS: 5-m walking speed; 6MWD: 6-min walking distance

### Effect of functional performance and NHI costs on PAC-CVD LOS

At week 3 of the PAC-CVD program, 55.3% of the variance in the PAC-CVD LOS was explained by the final regression model (*R*^2^ = 0.553, *F*_(15, 247)_ = 20.372, *p* < 0.001). The results of the hierarchical multiple regression analysis regarding the effects of functional performance and NHI costs on PAC-CVD LOS revealed that age, NHI costs, mRS score, and BI had significant effects; they contributed to 14.2% (confidence interval [CI]: −0.284 to − 0.049), *p* = 0.006), 38.6% (CI: 0.018 to 0.029, *p* < 0.001), 25.9% (CI: 1.760 to 7.075, *p* = 0.001), and 20.8% (CI: −0.219 to − 0.042, *p* = 0.004) of the regression model, respectively. Thus, the results revealed that old age was associated with a short LOS, whereas high NHI costs were associated with a long LOS. Moreover, a high mRS score, indicating severe disability, was linked to a long LOS, whereas a high BI, indicating high functional independence, was associated with a short LOS (Table 3).

**Table 3:** Effects of functional performance and NHI costs on LOS at week 3 (*N* = 263)

**Model Summary**										
Model	*R*	*R* ^2^	Adjusted *R*^2^		*R*^2^ change		*F* change		Sig. *F* change	Durbin-Watson
1	0.155	0.024	0.017		0.024		3.203		0.042	
2	0.215	0.046	0.008		0.022		0.736		0.659	
3	0.574	0.330	0.301		0.284		106.217		< 0.001	
4	0.744	0.553	0.526		0.223		30.821		< 0.001	0.768
**Predictive Factor Input**										
Model	Predictor			*B*		𝛽		95% CI		*p*
1	Constant			39.594				(29.530, 49.658)		< 0.001
	Age			-0.137		-0.118		(-0.280, 0.006)		0.060
	Gender			2.648		0.083		(-1.278, 6.574)		0.185
2	Constant			34.787				(20.384, 49.190)		< 0.001
	Age			-0.100		-0.086		(-0.263, 0.063)		0.227
	Gender			3.698		0.115		(-0.539, 7.936)		0.087
	Stroke type			1.737		0.051		(-3.303, 6.777)		0.498
	Previous stroke			2.260		0.059		(-2.500, 7.020)		0.351
	Hypertension			1.512		0.024		(-6.453, 9.478)		0.709
	Coronary artery disease			0.463		0.014		(-3.787, 4.713)		0.830
	Diabetes mellitus			-1.894		-0.061		(-5.806, 2.019)		0.341
	Hyperlipidemia			-2.210		-0.072		(-6.411, 1.991)		0.301
	Smoking			-0.916		-0.025		(-6.242, 4.409)		0.735
	Drinking			-0.838		-0.017		(-7.489, 5.814)		0.804
3	Constant			-28.817				(-45.965, -11.668)		0.001
	Age			-0.016		-0.013		(-0.153, 0.122)		0.824
	Gender			0.780		0.024		(-2.823, 4.383)		0.670
	Stroke type			1.702		0.050		(-2.532, 5.935)		0.429
	Previous stroke			0.363		0.010		(-3.652, 4.377)		0.859
	Hypertension			-1.291		-0.020		(-8.003, 5.421)		0.705
	Coronary artery disease			-0.142		-0.004		(-3.714, 3.429)		0.938
	Diabetes mellitus			-1.020		-0.033		(-4.311, 2.270)		0.542
	Hyperlipidemia			-1.431		-0.047		(-4.963, 2.101)		0.426
	Smoking			-1.295		-0.035		(-5.769, 3.178)		0.569
	Drinking			1.663		0.034		(-3.944, 7.270)		0.560
	Cost of NHI, USD			0.034		0.552		(0.027, 0.040)		< 0.001***
4	Constant			-4.885				(-25.388, 15.618)		0.639
	Age			-0.166		-0.142		(-0.284, -0.049)		0.006**
	Gender			0.296		0.009		(-2.720, 3.312)		0.847
	Stroke type			-0.158		-0.005		(-3.672, 3.357)		0.930
	Previous stroke			1.604		0.042		(-1.718, 4.926)		0.343
	Hypertension			-1.245		-0.020		(-6.852, 4.362)		0.662
	Coronary artery disease			0.015		< 0.001		(-2.964, 2.994)		0.992
	Diabetes mellitus			-0.475		-0.015		(-3.195, 2.246)		0.731
	Hyperlipidemia			0.359		0.012		(-2.572, 3.290)		0.810
	Smoking			-0.315		-0.009		(-4.024, 3.394)		0.867
	Drinking			0.971		0.020		(-3.658, 5.599)		0.680
	Cost of NHI, USD			0.023		0.386		(0.018, 0.029)		< 0.001***
	mRS			4.417		0.259		(1.760, 7.075)		0.001***
	BI			-0.130		-0.208		(-0.219, -0.042)		0.004**
	5MWS			1.348		0.040		(-3.681, 6.376)		0.598
	6MWD			-0.017		-0.143		(-0.036, 0.001)		0.070

Adjusted *R*^2^: adjusted goodness-of-fit measure for the regression model; *B*: unstandardized coefficient; 𝛽: standardized coefficient; NHI: national health insurance; mRS: modified Rankin scale; BI: Barthel index; 5MWS: 5-m walking speed; 6MWD: 6-min walking distance. Model 1 includes patient demographic factors (i.e., age and gender). Model 2 includes demographic factors, clinical attributes (i.e., stroke type, stroke history, hypertension, coronary artery disease, diabetes mellitus, and hyperlipidaemia), and common risk factors (i.e., smoking and drinking). Model 3 includes demographic factors, clinical attributes, common risk factors, and NHI costs. Model 4 includes demographic factors, clinical attributes, common risk factors, NHI costs, and functional performance (i.e., mRS score, BI, 5MWS, and 6MWD). Dependent variable: LOS in the PAC-CVD program. **p < 0.01. ***p < 0.001

At week 6 of the PAC-CVD program, 37.8% of the variance in LOS in the PAC-CVD program was explained by the final regression model (*R*^2^ = 0.378, *F*_(15, 136)_ = 5.501, *p* < 0.001). Age (CI: −0.331 to − 0.059, *p* = 0.005) and NHI costs (CI: 0.011 to 0.021, *p* < 0.001) had significant power to explain LOS in the PAC-CVD program. Thus, as age increases, LOS in the PAC-CVD program decreases; however, as NHI costs increase, this LOS increases (Table 4).

**Table 4:** Effects of functional performance and NHI costs on LOS at week 6 (*N* = 152)

**Model Summary**										
Model	*R*	*R* ^2^	Adjusted *R*^2^		*R*^2^ change		*F* change		Sig. *F* change	Durbin-Watson
1	0.152	0.023	0.010		0.023		1.766		0.175	
2	0.255	0.065	-0.001		0.042		0.790		0.613	
3	0.566	0.321	0.267		0.256		52.646		< 0.001	
4	0.615	0.378	0.309		0.057		3.118		0.017	0.571
**Predictive Factor Input**										
Model	Predictor			*B*		𝛽		95% CI		*p*
1	Constant			51.711				(42.591, 60.831)		< 0.001
	Age			-0.122		-0.153		(-0.251, 0.007)		0.064
	Gender			-0.957		-0.040		(-4.787, 2.872)		0.622
2	Constant			44.553				(30.866, 58.241)		< 0.001
	Age			-0.116		-0.146		(-0.274. 0.041)		0.147
	Gender			-0.275		-0.012		(-4.382, 3.833)		0.895
	Stroke type			2.308		0.098		(-2.439, 7.054)		0.338
	Previous stroke			4.141		0.153		(-0.376, 8.658)		0.072
	Hypertension			0.658		0.014		(-6.927, 8.242)		0.864
	Coronary artery disease			1.856		0.076		(-2.277, 5.988)		0.376
	Diabetes mellitus			2.194		0.097		(-1.625, 6.014)		0.258
	Hyperlipidemia			1.681		0.076		(-2.253, 5.614)		0.400
	Smoking			-1.616		-0.063		(-6.691, 3.460)		0.530
	Drinking			0.701		0.021		(-5.528, 6.930)		0.824
3	Constant			-22.393				(-44.070, -0.716)		0.043
	Age			-0.164		-0.206		(-0.300, -0.029)		0.018*
	Gender			-0.094		-0.004		(-3.609, 3.421)		0.958
	Stroke type			1.204		0.051		(-2.868, 5.276)		0.560
	Previous stroke			3.239		0.120		(-0.633, 7.112)		0.100
	Hypertension			2.148		0.047		(-4.354, 8.650)		0.515
	Coronary artery disease			2.052		0.084		(-1.484, 5.589)		0.253
	Diabetes mellitus			1.120		0.050		(-2.160, 4.401)		0.501
	Hyperlipidemia			2.693		0.122		(-0.684, 6.070)		0.117
	Smoking			-1.189		-0.046		(-5.533, 3.155)		0.589
	Drinking			-0.162		-0.005		(-5.497, 5.172)		0.952
	Cost of NHI, USD			0.018		0.516		(0.013, 0.023)		< 0.001***
4	Constant			-16.738				(-44.136, 10.659)		0.229
	Age			-0.195		-0.244		(-0.331, -0.059)		0.005**
	Gender			-0.035		-0.001		(-3.466, 3.395)		0.984
	Stroke type			0.452		0.019		(-3.597, 4.501)		0.826
	Previous stroke			3.750		0.139		(-0.080, 7.580)		0.055
	Hypertension			0.685		0.015		(-5.979, 7.350)		0.839
	Coronary artery disease			2.024		0.083		(-1.471, 5.519)		0.254
	Diabetes mellitus			0.924		0.041		(-2.292, 4.140)		0.571
	Hyperlipidemia			3.137		0.142		(-0.165, 6.440)		0.062
	Smoking			-0.828		-0.032		(-5.127, 3.470)		0.704
	Drinking			0.073		0.002		(-5.161, 5.306)		0.978
	Cost of NHI, USD			0.016		0.455		(0.011, 0.021)		< 0.001***
	mRS			2.168		0.144		(-1.577, 5.912)		0.254
	BI			-0.06		-0.011		(-0.127, 0.115)		0.925
	5MWS			-3.544		-0.109		(-11.900, 4.812)		0.403
	6MWD			-0.003		-0.025		(-0.032, 0.026)		0.853

Adjusted *R*^2^: adjusted goodness-of-fit measure for the regression model; *B*: unstandardized coefficient; 𝛽: standardized coefficient; NHI: National Health Insurance; mRS: modified Rankin scale; BI: Barthel index; 5MWS: 5-m walking speed; 6MWD: 6-min walking distance. Model 1 includes patient demographic factors (i.e., age and gender). Model 2 includes demographic factors, clinical attributes (i.e., stroke type, stroke history, hypertension, coronary artery disease, diabetes mellitus, and hyperlipidaemia), and common risk factors (i.e., smoking and drinking). Model 3 includes demographic factors, clinical attributes, common risk factors, and NHI costs. Model 4 includes demographic factors, clinical attributes, common risk factors, NHI costs, and functional performance (i.e., mRS score, BI, 5MWS, and 6MWD). Dependent variable: LOS in the PAC-CVD program. *p < 0.05. **p < 0.01. ***p < 0.001

At week 9 of the PAC-CVD program, the hierarchical multiple regression analysis demonstrated no significant effects on LOS in the PAC-CVD program (*F*_(15, 10)_ = 1.295, *p* = 0.346). Therefore, the regression model had no significant power to explain LOS in the PAC-CVD program.

## Discussion

This study is the first to investigate the effects of both functional performance and NHI costs (healthcare-related costs) on how long patients with stroke remain in the PAC-CVD program. The results indicated that in week 3 of hospitalization, age, NHI costs, mRS score, and BI were significant predictors of LOS. In week 6 of hospitalization, age and NHI costs remained significant predictors of LOS. However, in week 9 of hospitalization, patient demographics (age, gender), clinical attributes (stroke history, stroke type, hypertension, coronary artery disease, diabetes mellitus, and hyperlipidemia), common risk factors (smoking and drinking), NHI costs, and functional performance (mRS score, BI, 5MWS, and 6MWD) were not found to significantly predict LOS.

When an individual experiences a stroke in Taiwan, they are typically sent to either a medical centre or regional hospital for acute medical treatment. Once their condition stabilizes, they are discharged and must continue their recovery through outpatient rehabilitation at a small-scale hospital or clinic. However, an inability to continue rehabilitation at a medical centre often causes patients to feel helpless, and some may choose to transfer to another medical centre or large hospital for inpatient rehabilitation. Consequently, a high number of patients with stroke remain in large-scale hospitals for treatment, which leads to high NHI expenditure and consumes emergency and intensive care hospital beds and resources [13]. According to Taiwan’s NHI database, patients with stroke have long hospital stays (> 30 days) and are likely to require readmission. Approximately 10.4% of patients with stroke require long-term hospitalization in an acute care ward [24, 25]. Hospitals worldwide face similar challenges as they aim to maximize utilization of their limited bed capacity. Moreover, the demand for hospitalization remains high due to an increase in the older adult population. Shortening hospital stays is the conventional approach to addressing this problem, but such actions may jeopardize rehabilitation because patients may be discharged before they regain their physical function [26–28]. Therefore, the PAC-CVD program plays a crucial role in ensuring that patients with stroke can seamlessly transfer from a medical centre to a regional or district hospital for high-intensity rehabilitation and medical interventions during the postacute phase. The program enhances patients’ physical function and provides support for their return home or transfer to a long-term care institution. One study found that during 0–12 weeks of hospitalization in the PAC-CVD program, the functional performance of patients significantly improved [12]. However, longer hospitalization within the PAC-CVD program may result in community hospitals facing a shortage of PAC-CVD beds, which increases the wait time for beds or requires patients to be transferred to a hospital with available beds [13].

The results of this study indicate that mRS score and BI have significant power to explain LOS at week 3 of hospitalization. These two functional assessments evaluate a patient’s general disability level and independence in performing activities of daily living [15, 16]. Typically, patients with stroke who participate in the PAC-CVD program have weaker general motor abilities during the earlier stages of rehabilitation. During this period, the goals of rehabilitation are to improve patients’ overall functional abilities and reduce their dependence, which can subsequently affect LOS in the PAC-CVD program. Therefore, walking ability may not be the primary focus at this stage or a key consideration for LOS. Surprisingly, the functional performance measures (i.e., mRS score, BI, 5MWS, and 6MWD) at weeks 6 and 9 of hospitalization did not significantly explain the patients’ LOS. This finding may due to the PAC-CVD program’s policy of a typical 3–6 weeks of hospitalization, which results in most patients being discharged by week 6. If a patient requires inpatient rehabilitation for more than 6 weeks, the medical team must submit a team meeting record to apply to the NHIA for an extension. If the extension request is approved, hospitalization can be extended. The team meeting record must clearly document the assessments made by medical professionals, the patient’s condition and progress, the level of cooperation of the patient, and the effectiveness of rehabilitation. Therefore, the team meeting record strongly affects whether the NHIA approves the extension. Factors that may influence whether an extension is applied for at week 6 and how long the extension can be permitted vary greatly. The aforementioned factors are as follows: (1) Patient factors: an extension application is mainly initiated at the patient’s request, and the patient may consider aspects including the severity of the stroke, age, prestroke functional status, physical and cognitive function, comorbidities, social and family support, economic status, and the possibility of returning home [29]; (2) Medical team factors: opinions on whether the patient should continue to receive inpatient rehabilitation may differ between healthcare professionals depending on their expertise and knowledge. Clinicians focus on the patient’s medical stability and treatment for comorbidities; physical therapists concentrate on the patient’s overall motor status, balance, and muscle strength; occupational therapists focus on the patient’s activity of daily living functions, ability to participate in activities, and readiness to return to work; speech therapists concentrate on the patient’s ability to express and comprehend language and their swallowing function; social workers are most concerned with the patient’s family support, economic capacity, and social resources; dietitians concentrate on the patient’s dietary intake and nutritional needs; and finally, discharge preparation service workers focus on the patient’s placement after discharge, improving accessibility in their home, and applying for assistive devices; (3) Supervisory authority factors: following the submission of an application for a hospitalization extension, the NHIA determines the appropriate duration of further hospitalization on the basis of the patient’s assessments and the content of the team meeting records. If the extension is approved, the medical team continues providing inpatient rehabilitation. However, if the extension is rejected, the medical team prepares discharge and referral services accordingly; (4) Medical institution factors: in addition to running the PAC-CVD program, hospitals must care for other patients and flexibly use their limited medical resources and beds to ensure that patients in urgent need of acute treatment receive timely care. Moreover, hospitals must make preparations to discharge stable patients. Although the PAC-CVD LOS is influenced by the aforementioned various factors, Salbach et al. reported that for patients with stroke, 5MWS < 0.3 m/s may indicate that inpatient rehabilitation is required, whereas 5MWS ≥ 0.6 m/s indicates that a patient can return home [19]. In clinical settings, the medical team can predict the need for hospitalization and the discharge destination on the basis of the patient’s walking speed and develop a plan for continuing inpatient rehabilitation or discharge and referral assistance.

Regarding NHI costs, under the per-diem reimbursement system, the PAC-CVD program can effectively restore physical function without the patient incurring additional medical expenses [12]. The present study found that at weeks 3 and 6, NHI costs were significantly associated with the LOS recommended by the PAC-CVD program; thus, higher costs during this period corresponded to a longer stay. However, according to the multiple hierarchical regression model for week 9, neither functional performance nor NHI costs significantly explained LOS. Therefore, an extended LOS is not significantly related to NHI costs. Consequently, NHI costs are not significantly associated with LOS in the PAC-CVD program for patients for whom extension of hospitalization has been authorized. This lack of significance may suggest that the NHIA’s implementation of extended application strategies not only controls the LOS but also NHI costs.

Several studies have found that age is a factor influencing LOS in the PAC-CVD program, and our study yielded a similar finding; specifically, we discovered that LOS tends to be shorter for older patients than for younger patients [12, 30, 31]. Rehabilitation teams may have lower expectations regarding functional recovery in elderly patients. Although the PAC-CVD program provides high-intensity rehabilitation (3–5 sessions per day), older patients have lower exercise tolerance than do younger patients, which may increase the likelihood of early discharge. By contrast, older patients are more likely to experience complications such as urinary tract infection, pneumonia, or other infectious diseases during hospitalization, which can suspend the rehabilitation program and lead to a patient being transferred to an acute care unit. Furthermore, younger patients are considered to have longer life expectancy, be more productive, and be a greater source of household income. They also tend to have more ambitious rehabilitation goals than older patients, such as returning to work. Consequently, younger patients have a greater LOS to ensure they reach their rehabilitation goals [12, 30–34]. In 2019, Taiwan implemented a home-based PAC program that provided an alternative rehabilitation option for patients with stroke in the postacute stage and who were unable to receive hospital care but could still benefit from active rehabilitation. The results revealed that performing bedroom and bathroom tasks in a familiar home environment had several advantages. Furthermore, home-based PAC is more cost-effective than inpatient PAC for stroke rehabilitation [35]. Clinical healthcare professionals can offer home-based PAC programs to patients as an alternative to inpatient rehabilitation.

Although this study obtained valuable findings, it has some limitations. First, the sample was drawn from a single centre, which may limit the study’s generalizability. Future studies should recruit more patients across several centres. Second, to provide a tailored rehabilitation plan, different types of training were administered to individual patients. This may result in varying recovery durations that subsequently impact the LOS. Consequently, this can have implications for the overall cost of hospitalization within the NHI per diem reimbursement system. Third, psychological factors may have affected rehabilitation and LOS. Qualitative methods and a deeper exploration of the psychological factors are warranted. Finally, the statistical methods used in this study analysed the effect of functional performance and NHI costs on LOS at three assessment time points; however, because healthcare is patient-centred, individual patients could be assessed repeatedly to yield longitudinal data for investigating the effects on LOS. Despite these limitations, this study contributes to the literature by highlighting the effects of functional performance and medical costs on LOS in the PAC-CVD program.

## Conclusions

This study analysed the effects of functional performance and NHI costs on LOS in the PAC-CVD program for patients with stroke. At week 3, mRS score and BI significantly influenced this LOS, indicating that the degree of disability and independence in daily living activities during the early stage of hospitalization greatly affects the LOS. Clinical rehabilitation personnel can develop individualized treatment plans with such scores in mind. At week 6 and 9, functional performance did not significantly affect LOS in the PAC-CVD program. However, clinical personnel can devise inpatient rehabilitation plans or assist with transferring patients to outpatient care on the basis of their functional abilities such as walking speed. If hospitalization lasts less than 6 weeks, higher NHI expenditure corresponds to a longer LOS. If hospitalization is extended (> 6 weeks), NHI expenditure does not significantly affect the LOS. The NHIA’s policy of controlling hospitalization through extension applications may also be used to control medical expenses. However, the older the patient is, the shorter is their LOS. Although NHI expenditure is well-controlled, extended hospitalization for patients with declines in all functional measures may be discouraged in order to efficiently utilize limited PAC-CVD beds. Home-based rehabilitation can be an alternative to inpatient rehabilitation that improves patients’ activities of daily living would facilitate the return of individuals to their homes and help control NHI costs. Under the PAC-CVD policy, LOS is affected by various factors. The present study can be a valuable resource for medical decision-makers and clinical practitioners who devise medical care plans and arrange postdischarge referrals.

## Data Availability

The data presented in this study are available on request from the corresponding author.

## Abbreviations

CI: confidence interval
LOS: length of stay
NHI: National Health Insurance
NHIA: National Health Insurance Administration
mRS: modified Rankin scale
BI: Barthel Index
PAC-CVD: postacute care cerebrovascular disease
SD: standard deviation
VIF: variance inflation factor
5MWS: 5-m walking speed
6MWD: 6-min walking distance
